# Determinants of seasonal influenza vaccination in pregnant women in Valencia, Spain

**DOI:** 10.1186/s12889-016-3823-1

**Published:** 2016-11-21

**Authors:** R. Vila-Candel, P. Navarro-Illana, E. Navarro-Illana, E. Castro-Sánchez, Kiri Duke, F. J. Soriano-Vidal, J. Tuells, J. Díez-Domingo

**Affiliations:** 1 Midwifery at La Ribera Hospital Health Department, Carretera Corbera, km 1, 46600 Alzira, Valencia Spain; 2 Faculty of Nursing, Universidad Católica de Valencia” San Vicente Mártir”, c/ Espartero, 7. 46007 Valencia, Spain; 3 National Institute for Health Research Health Protection Research Unit (NIHR HPRU) In Healthcare Associated Infection and Antimicrobial Resistance at Imperial College London, Du Cane Road, London, W12 0NN UK; 4 Xàtiva-Ontinyent Health Department, Ausias March, 46.800 Xativa, Spain; 5 Cátedra de Vacunología Balmis, University of Alicante, Carretera de San Vicente del Raspeig, s/n. 03690, San Vicente del Raspeig, Alicante, Spain; 6 Vaccine Research, Fundación para el Fomento de la Investigación Sanitaria y Biomédica de la Comunitat Valenciana, FISABIO-Public Health, Avda. Catalunya, 21. 46020 Valencia, Spain

**Keywords:** Influenza Vaccines, Pregnancy, Acceptance, Influenza vaccine coverage

## Abstract

**Background:**

In most countries the coverage of seasonal influenza vaccination in pregnant women is low. We investigated the acceptance, reasons for rejection and professional involvement related to vaccine information in pregnant women in Valencia, Spain.

**Methods:**

Observational retrospective study in 200 pregnant women, 100 vaccinated and 100 unvaccinated, were interviewed during the 2014/2015 vaccination campaign. Electronic medical records, immunization registry and telephone interviews were used to determine reasons for vaccination and immunization rejection.

**Results:**

40.5% of pregnant women in the health department were vaccinated. The midwife was identified as source of information for 89% of women. The vaccine was rejected due to low perceptions of risk of influenza infection (23%), lack of information (19%), considering the vaccine as superfluous (16%), close proximity of delivery date (13%) and fear of side effects (12%).

**Conclusion:**

Pregnant women in Spain declined to be vaccinated due to under-estimation of the risk of contracting or being harmed by influenza, and lack of information. Interventions aiming to optimize vaccination coverage should include information addressing the safety and effectiveness of the current vaccine together with improved professional training and motivation.

## Background

Pregnancy is an independent risk factor for developing severe seasonal influenza [1]. Many international organizations recommend the administration of influenza vaccine to pregnant women [2, 3] since influenza infection is associated with higher maternal morbidity and mortality, increased hospital admissions and worse perinatal outcomes [4, 5].

Since 2004 influenza vaccination is recommended at any stage of pregnancy due to the well documented safety profile of the vaccine [6]. Despite its benefits, vaccination coverage among pregnant women remains low [7], according to international studies. In Spain there are no published data on influenza vaccination coverage in pregnant women, despite the clear benefits derived from close epidemiological surveillance of such coverage [8].

Different authors [1, 9] have highlighted that vaccination recommendation by health professionals is the main reason why women choose to be vaccinated against influenza. However, lack of information by health professionals also remains a frequently cited determining factor for rejecting vaccination [10]. Other studies have identified additional influences such as emotional or psychological factors (subjective emotional experiences, e.g. fear of side effects, doubts about the effectiveness of the vaccine, fear of needles/pain etc.) or under-estimation of personal risk (beliefs about the limited severity of the illness, influenza vaccination being unimportant, or non-association with important sequelae) [11–13] contributing to low vaccination rates.

Since a variety of studies have explored the scope of professional advice and its impact on vaccination acceptance, such evidence could be used to inform optimal strategies to improve vaccination coverage in this population at-risk [3, 14–16]. In Spain, pregnant women are entitled to receive free influenza vaccination.

The aim of the study was to investigate the acceptance of influenza vaccination amongst pregnant women in Valencia (Spain) and the reasons expressed for vaccination rejection. We also analyzed the knowledge that pregnant women had about the vaccine and described which healthcare professional was more frequently involved in the vaccination decision-making process during pregnancy.

## Methods

We conducted an observational, descriptive, retrospective study in women seen at *La Ribera* healthcare department in Valencia (Spain) for pregnancy follow-up and delivery between October 1, 2014 and January 31, 2015. This healthcare department has a *La Ribera* University Hospital (HULR), which is a tertiary healthcare center with 300 beds. The hospital and the healthcare department provide health services to 250,000 people approximately.

In the community (or primary care) we have family doctors, community nurses, community midwives and gynecologists as baseline care providers. One provider complements the other provider’s services and pregnant patients are followed up by the family doctor, the community midwife and the gynecologist.

During the study period there were 644 deliveries, of those 100 were not resident of the catchment area and 124 there was no information about the vaccination status in the vaccine registry Sistema de Información Vacunal (SIV) [17].

Of the remaining 420 women, 170 (40.5%) had received influenza vaccination. After reviewing the electronic charts we excluded women under 18 years, allergic to any of the vaccine components or women with communication barriers.

Of the remaining, we selected 100 women to be interviewed for each group (vaccinated and unvaccinated) through simple randomization sampling. Women were contacted by telephone in Feb-March 2015, and were interviewed after accepting a verbal informed consent.

We conducted a three-question telephone survey and asked vaccinated women about the source of the information of influenza vaccine, the health provider involved in recommending the vaccination, and whether they would opt to be vaccinated again in their next pregnancy (Table 1).

**Table 1:** Telephone questionnaire for vaccinated and unvaccinated women

Vaccinated women (*n =* 100)
1. Where did you receive information about the influenza vaccine while pregnant?
2. Which healthcare provider recommended the vaccine to you? If it was not a healthcare provider, who was it?
3. Would you choose to be vaccinated again in a future pregnancy under the same circumstances?
Unvaccinated women (*n =* 100)
1. Have you heard about the vaccine? If so, which healthcare provider recommended it to you?
2. Did you know whether you were eligible to be vaccinated?
3. Which was you reason for vaccine rejection?
4. Would you choose to be vaccinated again in a future pregnancy under the same circumstances?

We also conducted a four-question telephone survey on unvaccinated women, where we asked whether they had heard about the vaccine and, if so, which healthcare provider recommended it, the reasons for vaccine rejection, their knowledge about the vaccine and whether they would have the vaccine given in their next pregnancy (Table 1).

Other variables collected from the interview and the review of the electronic clinical notes included age, country of origin, gestational age at delivery, parity, and chronic underlying disease (asthma, diabetes, heart disease and immunosuppression).

The sample size was calculated to assess differences in the percentage of pregnant women that received advice from the health provider for influenza vaccination. We estimated that 50% of vaccinated women received advice and 40% of the unvaccinated. With an error of 5% and a power of 85%, the sample size needed was 194 women in total, 97 per group.

Bivariate analysis was performed using *Chi-square;* for risk factors for vaccination, an odds ratio (OR) with a 95% CI, was calculated. The significance level was set at *p* < 0.05.

### Ethics

The study was conducted according to the Declaration of Helsinki and current legislation, and received approval by the HULR Research Ethics Committee and the Spanish Medicines and Medical Devices Agency.

## Results

We contacted 141 vaccinated pregnant women, of those 41 (29%) did not participate: 35 (85.3%) did not answer the telephone, 4 (9.8%) declined to participate in the study and 2 (4.9%) were excluded due to communication difficulties. All of the vaccinated pregnant women confirmed their vaccination status, in agreement with the information on SIV.

On the other hand, 161 unvaccinated women were also contacted and 61 (37.9%) did not participate: 56 (91.8%) did not answer the telephone, 4 (6.6%) did not accepted participation and 1 (1.6%) was excluded due to communication barriers. All of the women with no information about the vaccination status in SIV confirmed having not received the vaccine.

The mean age was 31.5 ± 5.2 years (median 32.50, range 18–42), mean gestational age at delivery was 39.14 ± 1.5 weeks (range 34–42), and 47.2% (94/200) women were primiparous. The mean gestational age of vaccinated women was 32.28 ± 3.7 weeks (range 25–39).

The characteristics of both groups, vaccinated and unvaccinated are shown in Table 2, showing no statistical differences between them. Only 23% (3/13) of women with comorbidities (5.5 % asthma [11/200]; 0.5% diabetes [1/200]; 0.5% cardiac pathology [1/200]) were vaccinated, and 36% (36/100) women with at-risk pregnancy received vaccination. We observed that 40% (6/15) of the group who received the vaccine during the previous season (2013–14) rejected the vaccination during the pregnancy.

**Table 2:** Characteristics of pregnant women in study (*N =* 200)

		Vaccinated		Unvaccinated		
		(*n =* 100)		(*n =* 100)		
		n	%	n	%	*P-*value^a^
Country of Origin	Foreign	20	20.0	21	21.0	
	Spanish	80	80.0	79	79.0	0.831
Age (years)	<25	18	18.0	9	9.0	
	25–35	63	63.0	66	66.0	
	>35	19	19.0	25	25.0	0.143
Parity	1	51	51.0	43	43.0	
	2-mar	38	38.0	50	50.0	
	>3	11	11.0	7	7.0	0.201
Gestational	<37	9	9.0	10	10.0	
Age (weeks)	37–42	88	88.0	88	88.0	
	>42	3	3.0	2	2.0	0.881
Asthma^b^	No	97	97.0	92	92.0	
	Yes	34.0	3.0	8	8.0	0.121
Diabetes Mellitus^b^	No	100	100.0	99	99.0	
	Yes	0	0.0	1	1.0	0.316
Cardiac Pathology^b^	No	100	100.0	99	99.0	
	Yes	0	0.0	1	1.0	0.316
Immunosuppression^b^	No	100	100.0	100	100.0	
	Yes	0	0.0	0	0.0	N/A
Low Risk Pregnancy^c^	No	34	34.0	40	40.0	
	Yes	66	66.0	60	60.0	0.380
Pregnancy-induced Hypertension^c^	No	96	96.0	97	97.0	
	Yes	4	4.0	3	3.0	0.700
Thyroid Disease^c^	No	92	92.0	89	89.0	
	Yes	8	8.0	11	11.0	0.469
Smoker status^c^	No	84	84.0	79	79.0	
	Yes	16	16.0	21	21.0	0.363
Vaccinated previous season (2013–14)	No	91	91.0	94	94.0	
	Yes	9	9.0	6	6.0	0.421

^a^ *P*-value: based on chi-square test; N/A: insufficient cell number to perform chi-square analysis

^b^ Pre-gestational disease

^c^ Gestational disease or status during pregnancy

### Influenza vaccine acceptance

When vaccinated women were asked about the source of information regarding the vaccine, 98% (98/100) recalled being informed in their primary health care center. 1 % knew about it in advance as they were health professionals and another 1% received information via mass media such as radio, television or public health communication campaigns.

The information and recommendation of vaccination came mainly from their midwives (89%), in 9% (9/100) from the family doctor and 2% of women did not provide any information.

Of the vaccinated, 99% would have the influenza vaccine again in a future pregnancy should they find themselves in comparable health conditions.

### Influenza vaccine rejection

14% of women had not heard of influenza vaccination during pregnancy. Those who did have information had mainly received it from their midwife (*n =* 40) or other sources such as their family doctor (*n =* 6) or gynecologist (*n =* 1). Other information channels included public health advertising campaigns (*n =* 12), relatives or friends (*n =* 14) or at work (*n =* 2). Of the total sample, 6% (12/200) were health care professionals and knew about the vaccine, however, 91.6% (11/12) of these women declined to be vaccinated [OR = 13.1, 95% CI: 1.6–102.6; *p* = 0.02].

When asked if they were eligible to be vaccinated, 59% (59/100) responded positively, 4% (4/100) recognized they could not receive it at the time and 12% (12/100) did not know.

**Fig. 1:**
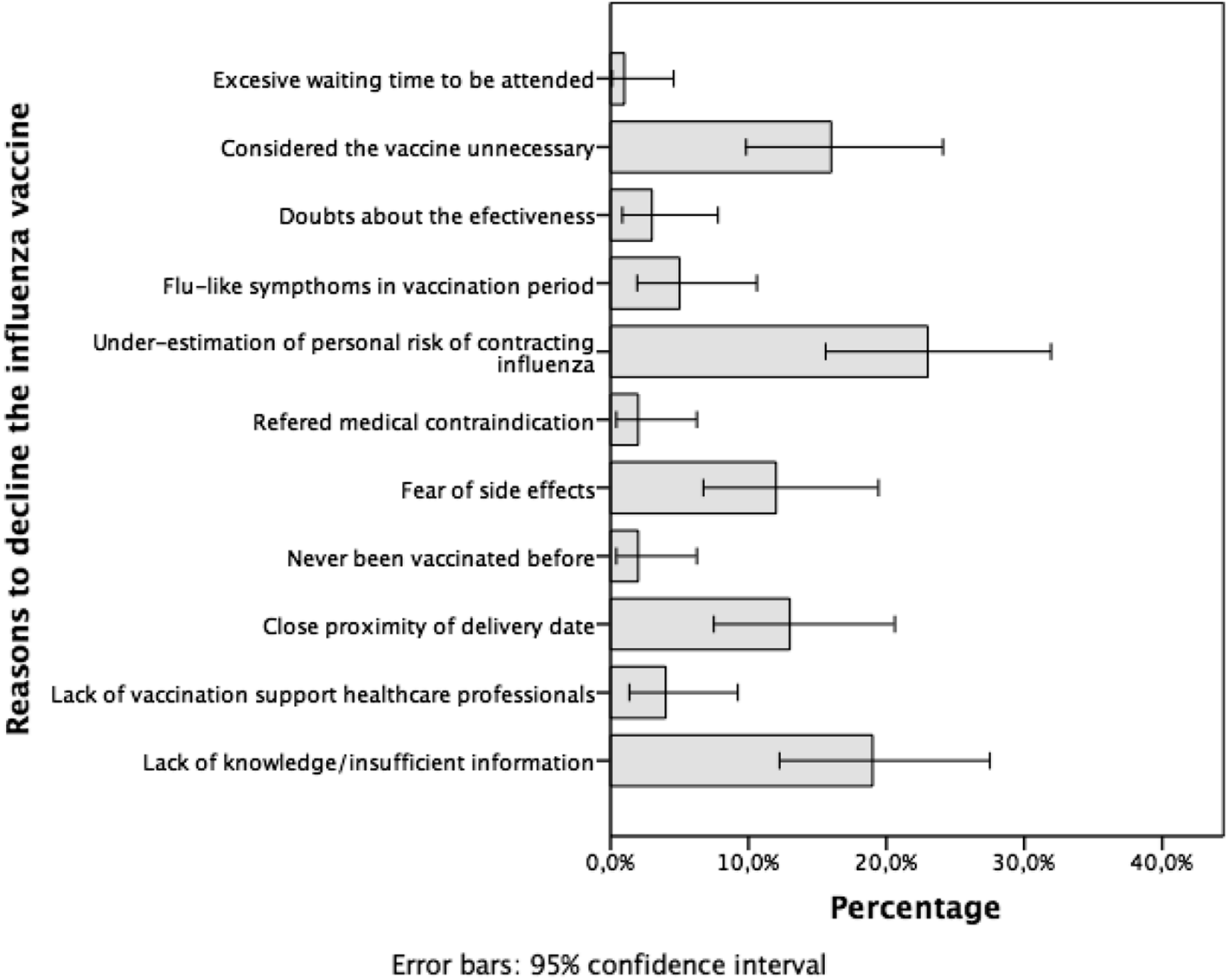
Reasons for influenza vaccine rejection during pregnancy

The reasons provided by women to decline the influenza vaccine during pregnancy are presented in Fig. 1. Women also stated that the main reason for rejecting vaccination was under-estimation of the risk, reflected in perceptions of no risk of contracting the illness whilst being pregnant (23%), followed by a lack of information (19%), considering the vaccine as non-essential (16%) and avoiding it due to the close delivery date (13).

When women were asked if they would opt to be vaccinated in a future pregnancy should they remain in similar health condition, 18% (18/100) accepted, 65% (65/100) would again reject it and 17% (17/100) did not know what to answer.

## Discussion

This study suggests that pregnant women do not vaccinate against influenza due to the low perception of risk and a lack of evidenced-based information provided by health professionals.

No significant differences were identified in the socio-demographic and obstetric characteristics between vaccinated and unvaccinated pregnant women. Seasonal influenza remains a global public health problem [18] and demands specific strategies and a particular focus on pregnant women as a high-risk group for seasonal flu [19, 20].

### Influenza vaccine acceptance

Regarding vaccination status before pregnancy, we observed that having previously received the flu vaccination was positively associated to acceptance during pregnancy [21]. However, a medical history involving a risk prior to pregnancy, such as having asthma, diabetes or a heart disease during pregnancy, was not associated with a higher influenza vaccination rate.

In relation to the knowledge of women who reported having received information about the vaccine, both vaccinated and unvaccinated women agreed that the midwife had been the main source of information, in agreement with a similar study conducted in the UK [22]. Although in other countries the family doctor has been reported as the main information agent [2, 16, 23], possibly owing to different healthcare models.

Concerning the intention to being re-vaccinated in a future pregnancy were the women to be in the same conditions, the surveyed women presented mixed responses. Virtually all vaccinated women agreed they would be vaccinated as well, while, in contrast, those unvaccinated would decline, coinciding with certain studies [24, 25]. As for unvaccinated pregnant women, we observed that those who were healthcare professionals were paradoxically much more likely to decline to be vaccinated, as seen in other studies [2, 26].

In all, reasons for *vaccination rejection* can be classified according to four themes:

1. Lack of knowledge and information
  The lack of knowledge due to insufficient information (19%), together with a lack of vaccination support from healthcare professionals (4%), accounted for 23% of vaccination refusal reasons, corroborating previous results [2, 10, 27, 28]. These findings highlight the need to improve influenza vaccination promotion activities conducted by healthcare professionals for pregnant patients [3]. We believe that information should be timely provided, within a meaningful discussion and a shared decision-making perspective [9, 11] that favors vaccine acceptance or rejection within an optimum time frame [25]. Ideally, this discussion should reflect the information on the causes, symptoms, side effects and the importance of the disease to support their decisions.
  However, the provision of accurate and quality information would be insufficient to ensure adequate health outcomes if the influence of health literacy of patients is not accounted for. Health literacy links knowledge and user skills in appropriate decision making related to health and social care [29]. A low health literacy level has been linked to poor health outcomes, including low vaccination rates [30]. In our study, unvaccinated women had accessed a greater number of information channels, compared with vaccinated women. This difference possibly reflects a need to confirm personal perceptions about the vaccine that would not fit or be accepted by the mainstream vaccination narrative [31], or that are not in agreement with the information received from each channel [25].
  Therefore, in order to increase the number of vaccinated pregnant women, public health strategies could take advantage of intervention synergies, combining efficient communication materials with information regarding immunization and vaccine safety, along with continuous education strategies for healthcare professionals. Since midwives already achieve the highest rate of influenza vaccination recommendation, and as main source of information according to women [9, 24], their leadership on the design, implementation and evaluation of these multiple interventions would seem logical [29].
2. Pregnant women feel the vaccine is unnecessary, ineffective and that they have a low perception of risk of influenza infection
  16% of women surveyed considered the vaccine unnecessary. Such idea may be supported by considering that the risk of contracting the disease whilst pregnant is non existent (23%). Pregnant women could also assume that an absence of influenza infection in the pre-pregnancy period could be related to their lifestyle, and since they had not changed it, the infection would also not occur during pregnancy either. Additionally, 3% of the women considered the vaccine to be ineffective, failing to realize the wider effect that an insufficient number of vaccinated women would have on the effectiveness of the vaccine at population level [32].
  Furthermore, 13% of surveyed women believed that vaccination was unnecessary due to the imminent childbirth date. Such position obviously ignores the benefits that acquired immunity would provide for the newborn after delivery [27], and that may result in a significant reduction in perinatal infections [6].
3. Health risk perceptions
  The side effects of the vaccine were a concern for 12% of women. This apprehension referred not only to their own health but also the babies’, and would explain the lack of vaccine acceptance during pregnancy. Such negative perceptions, however, do not seem to be endorsed by the existing on the link between vaccination and adverse perinatal and maternal outcomes [8, 32–34].
  In short, our results consistently confirm those obtained by other groups [1, 27, 35], where more than half of vaccination rejections stemmed from a perceived lack of data on the efficacy and safety of vaccine. Due to the determinant influence of these perceptions and beliefs, it is essential to carry out qualitative studies that would inform and facilitate improvement interventions.
4. Medical contraindications
  In 2% (2/100) of the cases the vaccine could not be administered due to transient and self-limited infectious processes, simply requiring a postponement of the inoculation [31, 32].

## Conclusions

Pregnant women in Spain declined to be vaccinated due to an under-estimation of the risk of contracting or being harmed by influenza and lack of information. In order to increase the acceptance of influenza vaccine it would be necessary to improve the information offered to women by all members of the multidisciplinary team and to integrate the therapeutic advice onto the care path for pregnant women.

## Abbreviations

HULR: Hospital Universitario de la Ribera
SIV: Sistema de Información vacunal (vaccination registry)
